# Associations between Hospital Quality Outcomes and Medicare Spending per Beneficiary in the USA

**DOI:** 10.3390/healthcare9070831

**Published:** 2021-07-01

**Authors:** Karyn Cook, Brent Foster, I’sis Perry, Christy Hoke, Dana Smith, Leanne Peterson, John Martin, Michael Korvink, Laura H. Gunn

**Affiliations:** 1 Department of Public Health Sciences, University of North Carolina at Charlotte, Charlotte, NC 28223, USA; karyncook79@gmail.com (K.C.); bfoste02@gmail.com (B.F.); iperry2@uncc.edu (I.P.); christyhoke77@gmail.com (C.H.); smida285@gmail.com (D.S.); leanner1227@gmail.com (L.P.); 2 School of Data Science, University of North Carolina at Charlotte, Charlotte, NC 28223, USA; 3 ITS Data Science, Premier, Inc., Charlotte, NC 28277, USA; john_martin@premierinc.com (J.M.); michael_korvink@premierinc.com (M.K.); 4 Faculty of Medicine, School of Public Health, Imperial College London, London W6 8RP, UK

**Keywords:** Medicare spending per beneficiary, Hospital Compare, multiresponse multinomial ordered probit, hospital quality outcomes

## Abstract

The cost of healthcare in the United States has increased over time. However, patient health outcomes have not trended with spending. There is a need to better comprehend the association between healthcare costs in the United States and hospital quality outcomes. Medicare spending per beneficiary (MSPB), a homogeneous metric across providers, can be used to evaluate the association between episodic Medicare spending and quality of care. Fifteen inpatient outcome measures were selected from Hospital Compare data among all (*n* = 4758) facilities and transformed to quintiles to ensure comparability across measures and to reduce the influence of outliers on the analysis. Both univariate and multiresponse multinomial ordered probit regression models were utilized across outcome domains to quantify associations between outcomes and spending. We found that MSPB was not associated with quality of care in most cases, adding evidence of a lack of outcome accountability among Medicare-funded facilities. Furthermore, worse outcomes were found to be associated with increased spending for some metrics. Policies are needed to align quality of care outcomes with the increasing costs of U.S. healthcare.

## 1. Introduction

The cost of healthcare in the United States has steadily increased over time [1]. More recently, healthcare costs have exceeded 17% of the total gross domestic product (GDP), increasing its share of GDP threefold over the last 50 years [2,3]. In relative terms, administrative costs for healthcare have been as large as 8%, compared to a high of 3% in similar countries [2]. For example, hospital costs amounted to over USD 410 billion in 2016, with Medicare and Medicaid assuming 66.3% of that cost [4]. Despite the increasing costs and high burden on the country’s GDP, in a study of eleven high-income countries, the United States performed near the bottom in regard to key health outcomes such as life expectancy, infant mortality, and obesity rates, with health outcomes consistently not reflecting spending [2,5].

Healthcare quality is arguably more important than healthcare costs, covering aspects of effectiveness, safety, a culture of excellence, and desired outcomes [6]. The U.S. government also has an interest in recognizing quality, reflected in the Agency for Healthcare Research and Quality annual quality reports [7,8]. The Institute for Healthcare Improvement defined the Triple Aim to jointly address the need for enhancing experience of care and improving population health, while lowering per capita costs [9]. Defining quality measures is crucial to be able to assess outcomes [10]. Lacking an appropriate evidence base, data collection and performance methods, specialty performance metrics, and reporting methods can be detrimental to improving quality [11]. Collecting data that do not capture the full picture of a quality improvement initiative could lead an implementation team to make incorrect extrapolations outside the realm of its scope. If information regarding costs and structural implementation is not captured, it could lead to a system-wide expansion of a specific performance improvement process that is very costly and inefficient, despite improving outcomes. This may also only address the needs of a small number of patients, potentially at the expense of larger, currently standardized practices that benefit all patients. Optimal local enhancements extrapolated globally do not necessarily translate into global enhancements.

Research on the associations between healthcare spending and quality has led to inconclusive results [12]. However, such associations may be outcome-dependent, and outcomes may relate more to homogeneous cost metrics. The Department of Health and Human Services ensures Medicare payments are tied to meeting minimum standards of quality or value [13]. However, how that effectively translates to quality of specific outcomes remains unclear. Other government entities also recognize the importance of cutting costs and assessing/enhancing quality of care. An interview provided by the director of the Office of Management and Budget documented that though spending varied across the country, institutions with higher spending did not achieve better outcomes, and oftentimes, the focus had been on quantity over quality [14]. Within health maintenance organizations, more profitable institutions have higher quality outcomes over time [15]. Fisher et al. suggest greater accountability for cost of care by incentivizing providers through more transparent quality measurements, without concern for volume or types of services [16].

High spending may be attributed to payers adopting costly technologies and treatment methods that may not be more effective than already established technologies or methods [17] or justify the increases in marginal costs per patient/treatment [14,17]. Most notably, the variation in pricing and competing motivations across and within healthcare organizations make it difficult to establish a connection between quality and cost [17,18]. Several authors have suggested focusing on waste reduction in order to reduce costs without sacrificing quality [17,19]. This can be achieved by reducing overtreatment of patients, which may also increase the quality of their treatment [17,20]. An additional cost-saving strategy involves enhanced and more efficient reporting systems for quality measures, especially since practices have spent as much as USD 15.4 billion per year on reporting [21]. Comprehending the relationship between quality of care and spending within a healthcare setting is crucial for hospitals, insurers, and governmental healthcare funders to improve quality of outcomes without unnecessary spending increases.

Through the Hospital Quality Initiative, the Centers for Medicare and Medicaid Services (CMS) seek to assist hospitals with improving their quality of care. Accordingly, the CMS provide public datasets through their Hospital Compare (recently renamed to Care Compare [22]) database, utilizing data collected from over 4000 Medicare-certified hospitals, as a resource for hospitals and consumers [23,24]. Healthcare consumers can use this database, which includes multiple outcome measures across facilities, to evaluate hospital quality performance before deciding what facility to use for their medical care [25]. Approximately every three years, the CMS review the influence of their endorsed measures on quality of care. These measures of care are widely accepted among healthcare providers, with over 90% of hospitals indicating that the CMS measures were clinically significant and exhibited enhanced care [26].

Medical spending depends on a large range of factors, such as geographic practice cost differences, wage differences, or disease- and patient-specific factors. Therefore, adjusting for those measures is paramount to assessing the links between spending and health outcomes. Medicare spending per beneficiary (MSPB) is a measure used to compare facilities by standardizing the cost per covered individual within the Medicare health insurance plan. This price-standardized and risk-adjusted measure homogenizes the Medicare-funded spending per beneficiary across an episode of care initiated from a hospital inpatient stay [27]. Therefore, it discounts the core factors driving cost differences, allowing for a clean measure of spending that is comparable across facilities. Hospitals and governmental funding agencies can use the CMS-endorsed MSPB measure as a source of determining their effectiveness with service expenditures [28]. In turn, hospitals could improve coordination of care across the continuum in conjunction with implementing quality control methods to achieve targets of efficiency in line with peer facilities.

With standardized measures of spending and quality of outcomes available, it is important to understand the association between spending and outcomes at the facility level across the United States. The relationship between cost (i.e., Medicare spending) and quality is bidirectional. Inpatient complications may lead to return visits to acute or ambulatory facilities, resulting in higher episodic spending, while higher utilization through improved coordination of care in the larger patient episode may have an impact on 30-day outcomes where the MSPB and quality episodes overlap (e.g., mortality). In order to explore this research question, this manuscript assesses the associations between medical spending, as reflected in the standardized MSPB measure, with a series of standardized national outcomes available through CMS Hospital Compare.

## 2. Materials and Methods

### 2.1. Data

CMS Hospital Compare data for 2019 corresponding to *n* = 4758 facilities were used in this study [29]. Metrics of outcome quality within Hospital Compare are organized under fourteen domains and further classified by groupings. MSPB is reported as a normalized score representing the amount that Medicare spends for an episode of care originating from a hospital index visit in comparison with other included hospitals across the nation. Fifteen highly used quality measures were selected for analysis; fourteen measures were chosen from the outcome domain type, and one was selected from the composite domain type [30]. These fifteen quality measures were grouped into three categories: (1) complications, deaths, and unplanned hospital visits; (2) patient safety; and (3) patient experience. In the complications, deaths, and unplanned hospital visits group were six hospital-acquired infection (HAI) measures, six 30-day mortality rates, and all-cause 30-day readmission rate. Patient safety was measured by the Patient Safety Indicator composite score (PSI-90), and patient experience was represented by the Patient Survey Hospital Consumer Assessment of Healthcare Providers and Systems (HCAHPS) summary star rating measure.

Within the complications, deaths, and unplanned visits group within this analysis, the 13 quality measures included: 30-day mortality associated with heart failure, acute myocardial infarction (AMI), coronary artery bypass graft, stroke, pneumonia, and chronic obstructive pulmonary disease (identified by the variables MORT 1–6 within Hospital Compare); overall 30-day readmission rate (identified by READM); and healthcare-acquired infections associated with catheter-urinary tract infection, central-line-associated bloodstream infection, colon surgery, abdominal hysterectomy, Methicillin-resistant Staphylococcus aureus (MRSA), and Clostridium difficile (C. diff) infection (identified by the variables HAI 1–6).

Cohort-specific risk-standardized 30-day mortality, a quality indicator established by the Yale Center for Outcomes Research and Evaluation (CORE), represents all-cause mortality within 30 days from discharge among patients who were discharged from the hospital [23]. The mortality measure adjusts for patient-level risk identified over a 365-day patient history. Higher mortality rates may indicate deficiencies in the effectiveness, safety, and timeliness of care [31].

Appropriate care provided for disease-specific conditions in the initial hospitalization can reduce hospital readmissions and healthcare costs [32]. The CMS estimate unplanned readmissions as unplanned visits to an acute care hospital within 30 days of hospital discharge [24]. Hospital-wide all-cause readmission measures were included in this study since America’s Health Rankings states that about 16% of elderly Medicare patients are readmitted to the hospital within 30 days, at a cost of USD 13,800 per readmission [33].

Each HAI measure reports infection using the standard infection ratio (SIR) for observed infection for patients in a bed. The Centers for Disease Control and Prevention (CDC) attributes increased hospital costs to HAIs, providing justification for inclusion of this measure in the analysis [24]. Furthermore, hospitals that implement quality of care initiatives to prevent infections should have lower HAI rates and decreased cost associated with care [34].

Unlike the aforementioned outcome measures, the Patient Safety Indicator Composite (PSI-90) is a composite score using a weighted average of the observed-to-expected ratio for several other health outcome measures, such as pressure ulcer rates or deep vein thrombosis [35]. Patient safety indicators as quality measures represent preventable defects in care. Unanticipated events, such as those included in the PSI-90 composite score, contribute to the escalated cost of care due to additional in-hospital treatments and/or longer hospital stays, as well as follow-up treatment [24].

Patient experience is also a quality outcome measure included in this study. The HCAHPS survey consists of 32 questions related to hospital care and is completed by patients following an inpatient stay. This measure provides the patient’s perspective in determining quality. Results are ranked on a Likert scale from 1 (worst) to 5 (best). The summary star rating for a facility (identified by the variable PAT_EXP) is derived by computing the mean average of 10 HCAHPS measure star ratings: hospital rating, willingness to recommend the hospital to others, hospital cleanliness, hospital quietness, nurse communication, doctor communication, discharge information, care transition, hospital staff responsiveness, and communication about medication [36].

### 2.2. Study Design and Statistical Analysis

Data for continuous outcome variables were transformed to categorical ordered quintiles to reduce the influence of outliers and provide a homogeneous parameter interpretation across outcomes, aligning outcome scales to that of the patient experience HCAHPS star ratings (ordinal ranking on a 1–5 scale). Imputation of missing MSPB was considered, but small differences in resulting sample sizes with respect to the complete case analyses did not justify the approach.

Five domain models were defined assessing the association of MSPB with (1) six healthcare-associated infections (HAI domain model), (2) six 30-day mortality rates (30-day mortality domain model), (3) 30-day readmission rate (30-day readmission domain model), (4) patient safety index (PSI-90; patient safety domain model), and (5) patient experience HCAHPS star rating (patient experience domain model). Only hospitals with complete within-domain outcome metrics were used to avoid outcome imputation and define homogeneous sets of facilities with all available domain-based outcome metrics. Additionally, missing outcomes are not due to lack of reporting but, more importantly, because those measures are not applicable for those facilities (e.g., a hospital will not report colon surgery outcome metrics if it does not perform colon surgeries).

A multiresponse multinomial ordered probit model [37] was used for the multi-outcome domains, and univariate versions [38] were used for the single-outcome domains. Since the primary analysis consists of assessing the statistical significance of MSPB with respect to each of the measurable outcomes across models/domains, a Bonferroni multiple testing correction was applied to preserve the target family-wise error rate of α = 0.05.

A sensitivity analysis was performed employing a single model combining all five domains—i.e., all 15 quality outcome measures—to assess the potential benefit of accounting for cross-outcome correlations among outcomes from different domains, as opposed to only within-domain correlations. R statistical software version 4.0.3 was used across all analyses.

## 3. Results

Table 1 includes the raw summary statistics for all variables. Upon removing facilities without complete measure data across domains, there was only a difference of three observations between imputing and removing missing MSPB (*n* = 495 vs. *n* = 492, respectively). With such a small difference, results using a complete case analysis are reported for the primary and sensitivity analyses.

### 3.1. Primary Analysis

Table 2 presents results of the multivariate multinomial ordered probit models assessing the association between MSPB and HAI outcomes (*n* = 680) and 30-day mortality outcomes (*n* = 982) in the multi-outcome domains, as well as the univariate models across single-outcome domains. Using a Bonferroni multiple testing corrected significance level of 0.0033 (across univariate and multivariate domains), MSPB was found to be significantly associated with MRSA bacterium HAI (HAI5) (*p* < 0.0001) and 30-day mortality among AMI (*p* = 0.0025) patients within the HAI and 30-day mortality quality domains, respectively. MSPB ratios are therefore positively associated with MRSA bacterium rates as well as its 30-day mortality rate among AMI patients.

Across the univariate domains, the estimated associations between MSPB and the 30-day readmissions (*n* = 4390), patient safety (*n* = 3210), and patient experience star rating (*n* = 3406) outcome quality domains are also included in Table 2. MSPB was found to be significantly associated with 30-day readmissions (*p* < 0.0001) and patient experience as measured by the HCAHPS star rating (*p* < 0.0001). A statistically significant positive association exists between MSPB and 30-day all-cause readmission rate; however, there is a significant negative association between MSPB and HCAHPS patient experience star rating.

### 3.2. Sensitivity Analysis

Results of the sensitivity analysis combining all five domains—i.e., all 15 quality outcome measures—into a single model to assess the potential benefit of accounting for cross-outcome correlations among outcomes from different domains are provided in Table 3. When all outcomes are assessed jointly regardless of domain, no significant associations were found. However, a much smaller set of hospitals (*n* = 492) report measures across all outcomes versus domain-specific analyses.

## 4. Discussion

Significant positive associations were found between Medicare spending per beneficiary and 30-day mortality rate among AMI patients, MRSA bacterium healthcare-associated infection rate, and 30-day all-cause readmission rate in the primary analysis. While the association between 30-day readmissions and MSPB is expected, as readmissions are included in the MSPB spend episode [39], it corroborates the assumption that a reduction in readmissions may lead to reduced episodic cost captured by the MSPB measure. Additionally, a strong negative association was found between MSPB and patient experience as measured by HCAHPS star rating. While most results were nonsignificant, those outcomes which have significant associations with MSPB suggest that adverse events such as MRSA and mortality during an index visit for patients with AMI within the 30-day post-discharge timeframe may be associated with higher overall episodic costs captured by the MSPB measure.

Some studies that have looked at the relationship between AMI mortality rates and Medicare spending found that mortality rates decreased as spending increased [40,41], which differs from the results of this analysis. Data were briefly reviewed to determine possible explanations for our findings. Disparities in AMI mortality outcomes may be exacerbated by structural differences in quality of care between urban and rural communities, such as access to specialist physicians instead of generalists [42]. Another study, which also found that rural Medicare patients had higher 30-day AMI mortality rates compared to urban patients, notes that rural patients were less likely than urban patients to receive the recommended treatment for AMI, such as aspirin, nitroglycerin, and heparin within particular time frames upon arrival to a facility [43].

Over three quarters of U.S. hospitals are paid under the Medicare Inpatient Prospective Payment System (IPPS) [44]. Hospitals paid under the IPPS receive a predetermined, fixed amount based on the average cost of treatment, per diagnosis, for all participating hospitals [45]. The cases are categorized into a diagnosis-related group to determine the base rate with some additional funding adjustments for teaching hospitals, high percentage of low-income patients, the use of new technology, transfers, and high-cost cases [44]. Hospitals paid under the IPPS are subject to a one-fourth reduction of the annual payment update if Hospital Inpatient Quality Reporting (IQR) Program requirements are not met for each fiscal year [46]. This is one way to ensure that they are managing effective and efficient quality programs. As of 2014, Medicare payments have been reduced to hospitals with poor performance in their rates of certain hospital-acquired conditions, including MRSA bacteremia [47]. Interestingly, our results indicate that MSPB is positively associated with rates of hospital-acquired MRSA. Although the CMS do not reimburse hospitals for treatment of hospital-acquired infections [48], it is possible that complications, such as those associated with MRSA bacteremia, lead to further treatment, thus increasing overall episodic costs. The aforecited study examining the relationship between hospital-acquired MRSA and healthcare costs outlined characteristics of patients who had a positive MRSA culture. These patients were more likely to have had surgery during the first 2 days of admission, to have had a longer inpatient stay, and to have incurred higher outpatient costs during the 12 months prior to admission. Based on these characteristics, these patients may be considered sicker and may have incurred higher treatment costs not related to hospital-acquired MRSA bacteremia, which may explain the results of the primary analysis.

Our study found a negative association between the HCAHPS summary star rating, which is a measure of patient satisfaction, and Medicare spending, in line with prior research [49]. A 2017 study by MacLeod et al. links both decreased patient satisfaction and increased spending to inadequate health literacy among elderly patients [50]. In their study, health literacy includes skills a patient needs to successfully follow instructions from their doctor or pharmacist, provide their medical history, self-manage their medical conditions, and coordinate their care. Their findings suggest increased costs are often due to lack of preventive care and poor self-management of chronic health conditions leading to increased emergency room and inpatient visits. Patient satisfaction among this group of patients may be lower because their health outcomes following utilization of the healthcare system are suboptimal.

### Strengths and Limitations

Measures are not always applicable to all facilities, which leads to varying sample sizes per outcome. While the results were grouped by domain in an effort to address this problem, there is still a large number of potentially smaller facilities for which results are not available in the larger outcome domains. The results are not generalizable to all hospitals due to the level of missingness of information, as well as the possibility that it is not missing at random. For example, smaller hospitals would not report surgical outcomes if no surgeries were performed.

Only claims for beneficiaries admitted to eligible acute care inpatient hospitals (paid through IPPS) during the period of performance are included in the calculation of the MSPB measure [51]. While this limits the MSPB measure calculation, it remains a homogeneous approach across facilities.

While other covariates could be of interest in risk adjusting quality outcomes, such as location (rurality), they are not necessary given the scope of this study. The primary research question relates to whether higher episodic spending as captured by the MSPB measure has a statistically significant association with quality outcomes. MSPB already accounts for financially justifiable measures such as wage or general costs, and factors such as rurality may explain but not necessarily justify worse outcomes.

Another potential unaccounted factor is how quality of outcomes relates to hospital size, type of hospital, location, and patient access to care. LaPointe describes the challenge of providing quality care with high-cost, low-volume services to patients with limited access to care, poor socioeconomic status, and greater health disparities [52].

While the granularity of this study is at the hospital level, further information at the patient level that is currently unaccounted for in the outcome measures may help to explain some of the hospital-level variability. Hence, hospital-level information may lead to different associations between costs and outcomes than complete patient-level information, if available at a national level.

Future research using data at the patient level would help to confirm the findings and account for other factors that may not be captured in hospital level outcomes, including a wide range of social determinants of health. Additionally, further research is needed to investigate causal factors behind the association between MRSA and MSPB given that they are not driven by misaligned incentive systems, since the CMS do not pay for treatment of hospital-acquired infections. Finally, further research is also needed to understand the association between patient satisfaction and MSPB, since our findings do not show an anticipated positive linkage between increased MSPB and greater patient satisfaction. If higher costs do not translate into greater patient satisfaction, there is evidence of room for efficiency improvements in spending.

## 5. Conclusions

Healthcare consumers, providers, and funders could benefit from a better understanding of (dis)connections between quality of care and episodic cost. Hospitals would be able to target those hospital-based outcomes associated with higher episodic cost. Patients would benefit from enhanced outcomes while consuming fewer resources within the continuum of care. The disconnect between higher spending and outcomes, in addition to spiraling overall healthcare costs and subpar healthcare outcomes, deserve further and deeper analysis and policy changes to support and adequately reward financial and healthcare efficiency.

## Figures and Tables

**Table 1:** Descriptive statistics across measures for *n* = 4758 facilities.

Identifier	Description	Mean	SD	Missing (%)
MORT1	Acute myocardial infarction	12.84	1.11	2489 (52.3%)
MORT2	Coronary artery bypass grafting	3.14	0.86	3753 (78.9%)
MORT3	Chronic obstructive pulmonary disease	8.54	1.11	1220 (25.6%)
MORT4	Heart failure	11.63	1.68	1268 (26.6%)
MORT5	Pneumonia	15.78	2.06	746 (15.7%)
MORT6	Stroke	13.84	1.48	2247 (47.2%)
PSI	PSI-90 composite	0.99	0.18	1548 (32.5%)
HAI1	Central-line-associated bloodstream infection	0.76	0.68	2772 (58.3%)
HAI2	Catheter-associated urinary tract infection	0.82	0.65	2485 (52.2%)
HAI3	Colon surgery	0.84	0.69	2900 (60.9%)
HAI4	Abdominal hysterectomy	0.92	0.90	4040 (84.9%)
HAI5	MRSA bacteremia	0.88	0.73	3048 (64.1%)
HAI6	*C. diff* infection	0.73	0.51	1670 (35.1%)
READM	30-day readmission	15.25	0.75	368 (7.7%)
PAT_EXP	HCAHPS star rating	3.23	0.87	1352 (28.4%)
MSPB	Medicare spending per beneficiary	0.99	0.09	1659 (34.9%)

**Table 2:** Results across domain-specific ordered probit models of association between MSPB and quality outcome measures.

Identifier	Description	Estimated Coefficient	Std. Error	*p*-Value
**Hospital-Acquired Infection Quality Domain (*n* = 680)**				
HAI1	Central-line-associated bloodstream infection	0.9137	0.7983	0.2524
HAI2	Catheter-associated urinary tract infection	−0.7414	0.8297	0.3716
HAI3	Colon surgery	−0.2305	0.7702	0.7647
HAI4	Abdominal hysterectomy	−0.3236	0.8106	0.6897
HAI5	MRSA bacteremia	3.5119	0.7991	<0.0001
HAI6	*C. diff* infection	0.8527	0.7872	0.2787
**30-day Mortality Quality Domain (*n* = 982)**				
MORT1	Acute myocardial infarction	2.1556	0.7127	0.0025
MORT2	Coronary artery bypass grafting	1.1052	0.7174	0.1234
MORT3	Chronic obstructive pulmonary disease	0.4319	0.7105	0.5432
MORT4	Heart failure	−1.8292	0.7065	0.0096
MORT5	Pneumonia	1.1473	0.7050	0.1037
MORT6	Stroke	−0.7075	0.7279	0.3311
**30-day Readmissions Domain (*n* = 4390)**				
READM	30-day readmission	4.9015	0.4193	<0.0001
**Patient Safety Domain (*n* = 3210)**				
PSI	PSI-90 composite	1.028	0.3586	0.0041
**Star Rating Domain (*n* = 3406)**				
PAT_EXP	HCAHPS star rating	−6.4361	0.5005	<0.0001

**Table 3:** Results of a multivariate multinomial ordered probit model assessing the association between MSPB and all 15 outcome measures across five quality domains (*n* = 492).

Identifier	Description	Estimated Coefficient	Std. Error	*p*-Value
MORT1	Acute myocardial infarction	0.9350	1.3918	0.5017
MORT2	Coronary artery bypass grafting	0.7724	1.3669	0.5720
MORT3	Chronic obstructive pulmonary disease	0.5055	1.3730	0.7128
MORT4	Heart failure	0.2552	1.4019	0.8556
MORT5	Pneumonia	0.7497	1.3777	0.5864
MORT6	Stroke	0.4164	1.4163	0.7688
PSI	PSI-90 composite	1.0008	1.3949	0.4731
HAI1	Central-line-associated bloodstream infection	1.4527	1.3351	0.2766
HAI2	Catheter-associated urinary tract infection	1.0091	1.2890	0.4337
HAI3	Colon surgery	0.9605	1.3586	0.4796
HAI4	Abdominal hysterectomy	0.7242	1.3770	0.5989
HAI5	MRSA bacteremia	1.8069	1.3526	0.1816
HAI6	*C. diff* infection	1.6597	1.3257	0.2106
READM	30-day readmission	1.8058	1.3903	0.1940
PAT_EXP	HCAHPS star rating	0.6098	1.5185	0.6880

## Data Availability

Data are available from CMS’ Hospital Compare through the corresponding cited references.
